# Essential Oils from Annonaceae Species from Brazil: A Systematic Review of Their Phytochemistry, and Biological Activities

**DOI:** 10.3390/ijms222212140

**Published:** 2021-11-09

**Authors:** Márcia Moraes Cascaes, Odirleny dos Santos Carneiro, Lidiane Diniz do Nascimento, Ângelo Antônio Barbosa de Moraes, Mozaniel Santana de Oliveira, Jorddy Neves Cruz, Giselle Maria Skelding Pinheiro Guilhon, Eloisa Helena de Aguiar Andrade

**Affiliations:** 1 Programa de Pós-Graduação em Química, Universidade Federal do Pará, Rua Augusto Corrêa S/N, Guamá, Belém 66075-900, PA, Brazil; giselle@ufpa.br (G.M.S.P.G.); eloisa@museu-goeldi.br (E.H.d.A.A.); 2 Faculdade de Química, Universidade Federal do Pará, Rua Augusto Corrêa S/N, Guamá, Belém 66075-900, PA, Brazil; lenny_carneiro@hotmail.com (O.d.S.C.); angeloquimica17@gmail.com (Â.A.B.d.M.); 3 Laboratório Adolpho Ducke–Coordenação de Botânica, Museu Paraense Emílio Goeldi, Av. Perimetral, 1901, Terra Firme, Belém 66077-830, PA, Brazil; lidianenascimento@museu-goeldi.br (L.D.d.N.); jorddynevescruz@gmail.com (J.N.C.)

**Keywords:** natural products, Brazilian species, essential oil, applications

## Abstract

The present work involves a systematic review of the chemical composition and biological effects of essential oils from the Annonaceae species collected in Brazil from 2011 to 2021. Annonaceae is one of the most important botanical families in Brazil, as some species have economic value in the market as local and international fruit. In addition, the species have useful applications in several areas—for instance, as raw materials for use in cosmetics and perfumery and as medicinal plants. In folk medicine, species such as *Annona glabra* L. and *Xylopia sericea* A. St.-Hil. are used to treat diseases such as rheumatism and malaria. The species of Annonaceae are an important source of essential oils and are rich in compounds belonging to the classes of mono and sesquiterpenes; of these compounds, α-pinene, β-pinene, limonene, (*E*)-caryophyllene, bicyclogermacrene, caryophyllene oxide, germacrene D, spathulenol, and β-elemene are the most abundant. The antimicrobial, anti-inflammatory, antileishmania, antioxidant, antiproliferative, cytotoxic, larvicidal, trypanocidal, and antimalarial activities of essential oils from the Annonaceae species in Brazil have been described in previous research, with the most studies on this topic being related to their antiproliferative or cytotoxic activities. In some studies, it was observed that the biological activity reported for these essential oils was superior to that of drugs available on the market, as is the case of the essential oil of the species *Guatteria punctata* (Aubl.) R. A. Howard., which showed a trypanocidal effect that was 34 times stronger than that of the reference drug benznidazol.

## 1. Introduction

The species of Annonaceae are flowering plants consisting of trees, shrubs, and lianas. These species present a combination of striking characteristics and form one of the most uniform botanical families from both anatomical and structural points of view; they are one of the most primitive families of Angiosperms and belong to the class Magnoliopsida, subclass Magnoliidae, and order Magnoliales [1].

Annonaceae consists of 2106 species and more than 130 genera. The family is concentrated in the tropics, and about 900 species are neotropical, 450 are Afrotropical, and the others are Indomalayan [2]. Annonaceae plays an important ecological role in terms of species diversity, especially in tropical forest ecosystems [3]. In Brazil, the family has confirmed occurrence in all states, with about 380 species being described here, distributed across 32 genera. The Amazon biome contains three quarters of all Annonaceae species, with 268 occurring here. Meanwhile, the Atlantic Forest houses 98 species and the Cerrado has approximately 52 [4].

Some species of Annonaceae are of economic value in the international fresh fruit market, such as *Annona cherimola* Mill. (“Cherimólia”) and *Annona squamosa* L. (“pinha”) [5]. In Brazil, some *Annona* fruits are highly sought after, such as *Annona crassiflora* Mart. (“Araticum”), *A. squamosa*, and *Annona muricata* L. (“graviola”) [3]. In addition, some species are often used as raw materials in the cosmetics and perfumery industries and as medicinal plants [6].

Numerous species of Annonaceae are odoriferous and these fragrances are due to the presence of essential oils (EOs) [7]. In nature, EOs have many important functions, such as attracting insects or allowing allelopathic communication between plants [8]. In addition, they have antibacterial, antiviral, anti-inflammatory, and antifungal properties, among others [9].

According to the review published by Fournier et al. [10], the main volatile constituents of EOs from the Annonaceae species are monoterpene hydrocarbons in fruits and seeds, sesquiterpene hydrocarbons in leaves, and oxygenated sesquiterpenes in bark and roots. After this review (1999), several articles were made available in the literature showing the chemical and biological properties of EOs obtained from Annonaceae species [11,12,13,14]. In this context, the present work aims to carry out a systematic review of the essential oils of the Annonaceae species collected in Brazil in the last ten years, evaluating their chemical compositions and their potential biological activities.

## 2. Essential Oils

EOs are present in various aromatic plants, usually found in tropical and subtropical countries. They are obtained from various parts of aromatic plants, including leaves, flowers, fruits, seeds, buds, rhizomes, roots, and bark [9]. Chemically, EOs are mixtures of 20–60 components in varying concentrations, with some compounds found in high concentrations (20–70%) and others in only small amounts. Most of the components of EOs are designated as lipophilic terpenoids, phenylpropanoids, or derivatives of short-chain aliphatic hydrocarbons of low molecular weight, with the first being the most frequent and characteristic constituents. Among these, acyclic mono- and sesquiterpenoids and mono-, bi-, or tricyclics of different chemical classes constitute the majority of EOs, such as hydrocarbons, ketones, alcohols, oxides, aldehydes, phenols, and esters [15].

Several techniques have been used to obtain EOs. These techniques depend on the part of the plants from which the oil will be extracted, the stability of the oil when faced with heat, and the susceptibility of the oil’s constituents to undergoing chemical reactions. Some of the techniques commonly used for EO extraction are hydrodistillation, hydrodiffusion, enfleurage, cold pressing, steam distillation, solvent extraction, microwave-assisted process, and carbon dioxide extraction [16,17,18,19,20,21].

Essential oils play an important role in plants and act as antibacterials, antivirals, antifungals, and insecticides and protect plants from herbivores. It is possible to list about 3000 EOs, but only 300 are used in perfumes, and makeup products, sanitary products, dentistry, and agriculture; as preservatives and flavor additives for food; as fragrances for household cleaning products; as industrial solvents; and as natural remedies [22].

In recent years, EOs have gained great popularity in the food, cosmetic, and pharmaceutical industries. Consumers have developed increasing interest in the use of natural products as alternatives to artificial additives or pharmacologically relevant agents.

Medical professionals are more interested in the medicinal properties of EOs, as research has shown the antibacterial, fungicidal, relaxant, stimulant, and antidepressant effects of these volatile substances. Furthermore, EOs are known for their therapeutic properties and are therefore used in the treatment of various infections caused by pathogenic and non-pathogenic diseases [16].

Consumer concerns about chemical preservatives have driven the growing interest in some natural antimicrobials, such as EOs [23]. In the food industry, the current trend to reduce the use of food preservatives in favor of natural alternatives makes EOs and their components viable alternatives for this application [24].

In the food industry, limonene monoterpene, a component of many EOs, is used as a flavoring in the production of desserts, ice cream, and non-alcoholic beverages. Thymol, a crystalline substance with an intense odor that is part of the chemical composition of the EO of thyme (*Thymus vulgaris* L. and *T. zygis* L.) and whose content varies between 22% and 50%, is used as a flavoring agent in food products, such as sweets, syrups, and seasoning mixtures [25]. The monoterpene eucalyptol (or cineole) is a colorless liquid with a camphor odor; one of the most abundant sources of 1,8-cineole is *Eucalyptus globulus* Labill. leaves. The EO of this species is used as a flavoring additive in various food products (such as in meat) as well as in beverages. Due to the fresh odor of cineole, this substance is applied in large quantities in oral care products [25].

In the cosmetics industry, EOs are vital assets, as in addition to providing pleasant aromas for various products they are also able to act as preservatives and active agents and, simultaneously, offer several benefits to the skin. EOs with a high added value include are citrus, lavender, eucalyptus, tea tree, and other floral oils, which are used as fragrances, while linalool, geraniol, limonene, citronellol, and citral are very popular fragrance components used in different cosmetics [26].

## 3. Annonaceae Ethnobotanics

Natural products, especially those derived from plants, have been used to help humanity treat various ailments for many millennia [27]. Plants have played an important role in the survival of many human communities. They have been used in many different ways—e.g., as food, medicines, and ornaments; for mystical and religious purposes; as lumber; and for making handicrafts. Knowledge about the use of plant resources has been transmitted from father to son and from ancient civilizations to the present day.

Brazil is formed from the Amazon Forest biome, the Pantanal, savannah and woodland Cerrado, the semi-arid forest Caatinga, pampas fields, and the Atlantic Forest rainforest. These varied biomes reflect the enormous wealth of flora and the greatest biodiversity on the planet. In addition, there is great cultural diversity in Brazil and the use of medicinal plants results from different knowledge built over time [28].

The Amazon region has approximately 55,000 species of plants, most of which are still little known and many of which are used for medicinal and religious purposes [29]. In this region, indians, caboclos, riverside dwellers, rubber tappers, quilombolas, fishermen, small rural producers, and extractivists hold rich knowledge about plants that is passed from generation to generation through oral tradition. It is also known that the cultural diversity of this country positively influences the ethnobotanical use of medicinal plants and in a way increases its biodiversity, given the inclusion of exotic species in the national flora brought by the different peoples who have come to this country [30].

In this context, ethnobotany is a branch of science that analyzes and studies the knowledge of various peoples about the use of plants. It is through this that the profile of a community and its uses of plants are learned, as each community has its own customs and peculiarities, aiming to extract information that may be beneficial regarding the uses of medicinal plants [30].

Several plants of the Annonaceae family are used in folk medicine due to their pharmacological properties, which are attributed to the presence of secondary metabolites of different classes, such as alkaloids, acetogenins, and flavonoids [31].

The *Annona muricata* L. species, similar to other *Annona* species, including *A. squamosa* L. and *A. reticulata* L., are widely used in traditional medicine against a variety of diseases, especially cancer and parasitic infections. The fruits of *A. muricata* are used as a natural remedy for diseases such as neuralgia, arthritis, diarrhea, dysentery, fever, malaria, parasites, rheumatism, skin rashes, and worms. In addition, many women eat the fruit to increase their production of breast milk after giving birth. The leaves of this species are used to treat cystitis, diabetes, headaches, and insomnia. Extract made from the leaves has anti-rheumatic and neurologic effects, while the cooked leaves are used to treat abscesses and rheumatism. Crushed seeds are believed to be anthelmintic [32].

Among the medicinal species of *Xylopia*, *X. frutescens* Aubl. is found in Central and South America, Africa, and Asia. In Brazil, the plant is popularly known as ‘‘embira’’, ‘‘embira-vermelha’’, and ‘‘pau carne’’, and its seeds are used in folk medicine as a bladder stimulant; to trigger menstruation; and to combat rheumatism, halitosis, caries, and intestinal diseases [33]. The leaves and flowers of the *X. laevigata* (Mart.) R.E.Fr. species are used to treat painful diseases, heart disease, and inflammatory conditions [34]. *Xylopia sericea* A. St.-Hil. is an aromatic plant popularly known as pindaíba, pindaíba vermelha, and/or pimento-de-macaco; it is traditionally used as food and as an antimalarial, similar to other representatives of the *Xylopia* genus [35].

Some species of *Guatteria* are used in traditional medicine; in Northern Brazil, the seeds of *G. ouregou* (Aubl.) Dunal are used to treat dyspepsia, stomach pain, and uterine pain [36].

The species *Duguetia furfuracea* (A.St.-Hil.) Saff. is known as “araticum-seco”. In folk medicine, the powder from its seeds is mixed with water for use in the treatment of pediculosis, while an infusion of its leaves and branches is used to treat rheumatism. [37]. *Duguetia lanceolata A.St.-Hil.*, popularly known as pindaíba, beribá, or pinhão, is a perennial species distributed across several states of Brazil; in popular medicine, this plant has been used as an anti-inflammatory, for healing, and as an antimicrobial agent [38]. Table 1 shows the 19 species of Annonaceae used in traditional Brazilian medicine.

## 4. Phytochemistry of Annonaceae Essential Oils

The chemical composition of Annonaceae EOs is varied; in general, mono and sesquiterpenes are the most abundant compounds. In this work, were gathered studies on the chemical composition of EOs from Annonaceae, referring to 38 different species with a geographic distribution in the Brazilian territory. Information regarding the major chemical constituents identified (>5%), yield, collection data, and extraction method used for these EOs is shown in Table 2.

**Figure 1:**
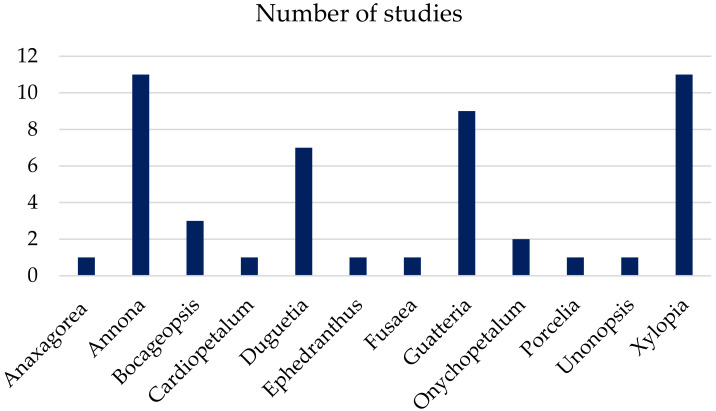
Distribution of studies with essential oils according to the genus of Annonaceae occurring in Brazil from 2011 to 2021.

**Figure 2:**
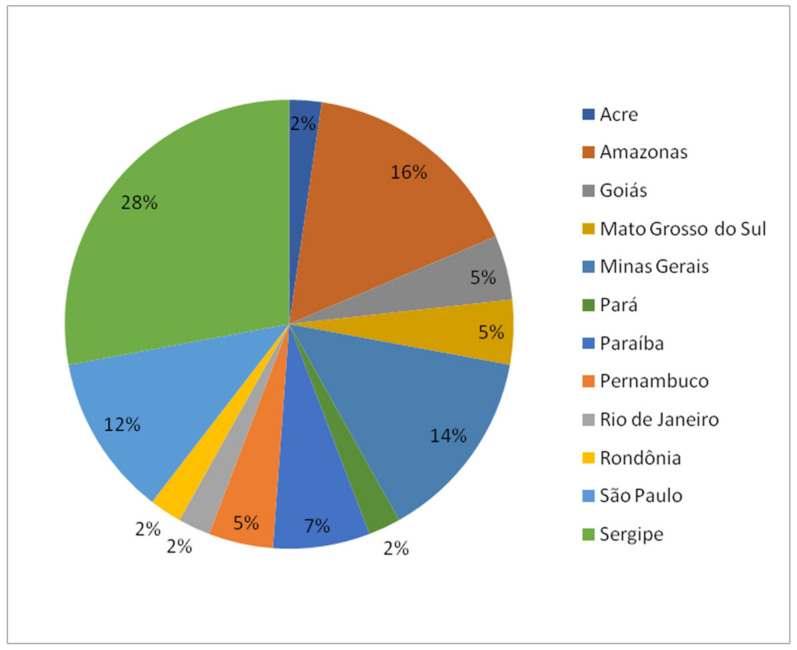
Percentage of studies conducted with essential oils from Annonaceae species collected in Brazil between the years 2011 and 2021.

**Figure 3:**
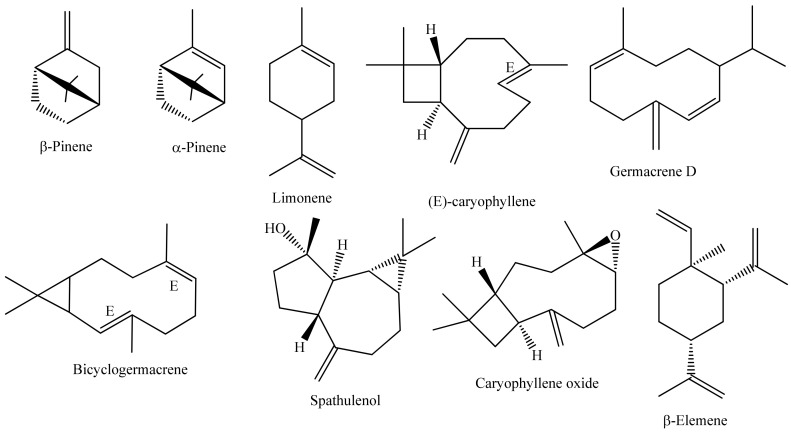
Structure of the major chemical constituents identified in the essential oils of Annonaceae species occurring in Brazil.

Most studies carried out with EOs of Annonaceae occurring in Brazil, published between the years 2011 and 2021, were conducted with species belonging to the genera *Annona*, *Guatteria*, and *Xylopia* (Figure 1). Collections were mostly carried out in the states of Amazonas and Sergipe (Figure 2). To date, about 100 volatile chemical constituents (>5%) have been obtained from the EOs of Annonaceae species collected in Brazil. Among these compounds, α-pinene, β-pinene, limonene, (*E*)-caryophyllene, bicyclogermacrene, caryophyllene oxide, germacrene D, spathulenol, and β-elemene are the most abundant (Figure 3).

## 5. Biological Activities

It is generally accepted that chemical composition determines the bioactivities of EOs. Annonaceae species have been widely used in folk medicine. Their EOs have been evaluated for several effects, including anti-inflammatory, antitumor, antibacterial, and antioxidant effects [49,61].

**Figure 4:**
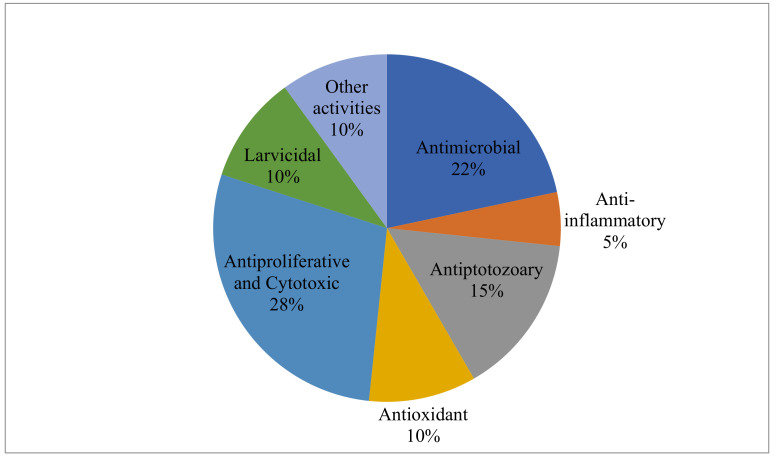
Distribution of studies on the biological activities of essential oils from Annonaceae species occurring in Brazil.

A total of 60 studies involving the biological activities of EOs from Annonaceae species collected in the Brazilian territory between the years 2011 and 2021 are described in this work. The bioactivities reported for Annonaceae EOs are represented in Figure 4. Several EOs presented more than one reported biological activity, with the most frequent studies being related to antiproliferative or cytotoxic activities, representing 28% of the results listed here.

### 5.1. Antimicrobian Activity

Annonaceae species are an important source of new antimicrobial agents for combating resistant microorganisms; several EOs of this family had their antimicrobial properties evaluated and showed potentially relevant results.

The EOs of *Bocageopsis multiflora*, *Duguetia quitarensis*, *Fusaea longifolia*, and *Guatteria punctata* were evaluated to determine their antibacterial activity [57]. The EO of *B. multiflora*, which is rich in *cis*-linalool oxide (furanoid) (33.1%) and 1-*epi*-cubenol (16.6%), showed antibacterial activity against Gram-negative and Gram-positive strains, with MIC values of 4.68 µg.mL^−1^. The EO of *D. quitarensis*, which is mainly composed of 4-heptanol (33.8%), α-thujene (18.4%), (*E*)-caryophyllene (14.4%), germacerne D (6.3%), and α-copaene (5.3%), was found to be active against the Gram-positive microorganisms *Streptococcus mutans* and *Streptococcus pyogenes*, with an MIC value of 37.5 µg.mL^−1^. The EO of *F. longifolia*, which is rich in β-selinene (19.3%), *cis*-β-guaiene (18.3%), (*Z*)-α-bisabolene (12.0%), and (*E*)-caryophyllene (7.1%), was found to be active against *Pseudomonas aeruginosa*, *Streptococcus mutans*, and *Staphylococcus aureus* and resistant to methicillin, with an MIC value of 37.5 µg.mL^−1^. The EO of *Guatteria punctata*, with a high content of germacrene D (19.8%), (*E*)-nerolidol (9.9%), (*E*)-caryophyllene (8.4%), and *cis*-β-guaiene (5.5%), was found to be active against *S. mutans* and *S. pyogenes*, with an MIC value of 4.68 µg.mL^−1^ [57].

The EO from the leaves of *Anaxagorea brevipes*, composed mainly of β-eudesmol (13.16%), α-eudesmol (13.05%), γ-eudesmol (7.54%), and guaiol (5.12%), showed an antibacterial and antifungal inhibitory effect against *Kocuria rhizophila*, penicillinase-negative *Staphylococcus aureus*, *Candida albicans,* and *Candida parapsilosis*, with MIC values ranging from 25.0 to 100.0 μg·mL^−1^ [51].

The EOs of *Xylopia aromatica*, which is rich in spathulenol (21.5%), dihydrocarveol (11.6%), and *trans*-pinocarveol (10.2%), as well as *Guatteria blepharophylla*, which is rich in caryophyllene oxide (55.7%), spathulenol (8.9%), and palustrol (6.5%), showed strong activity against the Gram-positive bacteria *Streptococcus sanguinis* (MIC = 0.02 mg.mL^−1^) [49].

The EOs from the leaves, branches, and bark of the trunk of *Onychopetalum amazonicum* were evaluated to determine their antimicrobial activity against four bacterial strains and five pathogenic fungi. The EO from the trunk bark exhibited activity against *Staphylococcus epidermidis*, *E. coli*, and *Kocuria rhizophila*, with an MIC value of 62.5 μg·mL^−1^. The observed activity may be associated with the presence of the sesquiterpene *allo*-aromadendreno (21.2%) [69].

The antibacterial activity of the EO of *Xylopia sericea* fruits was investigated and the results showed that this EO, which has a high content of the sesquiterpenes spathulenol (16.42%), guaiol (13.93%), and germacrene D (8.11%), has bacteriostatic effects against *S. aureus* (MIC = 7.8 µg.mL^−1^), *Enterobacter cloacae* (MIC = 7.8 µg.mL^−1^), *Bacillus cereus* (MIC = 15.6 µg.mL^−1^), and *Klebsiella pneumoniae* (MIC = 62.5 µg.mL^−1^) [50].

The antimicrobial activity of EOs from *Xylopia aromatica* flowers and leaves was tested against Gram-positive and Gram-negative bacterial strains and fungi. The EO of the flower, which is rich in pentadecan-2-one (16.38%), bicyclogermacrene (9.74%), 7-*epi*-α-eudesmol (7.76%), khusinol (7.23%), *n*-tricosane (6.17%), and heptadecan-2-one (5.83%), and the EO of the leaf, which is rich in spathulenol (27.11%), khusinol (13.04%), bicyclogermacrene (8.52%), globulol (6.47%), and *cis*-guaia-3,9-dien-11-ol (5.98%), exhibited a lower MIC against *S. pyogenes* (200 and 100 µg.mL^−1^, respectively) [73].

The EOs from two specimens of *Guatteria elliptica* collected in Paranapiacaba and Caraguatatuba (São Paulo), which have high levels of spathulenol (53.9%) and caryophyllene oxide (40.9%), respectively, showed an inhibition of growth of less than 100% at the highest concentration tested (3 mg.mL^−1^), and MIC values > 3 mg.mL^−1^ against all the microorganisms tested [9].

The EOs from four *Guatteria* species (*G. australis, G. ferruginea, G. latifolia*, and *G. sellowiana*), which are rich in spathulenol (11.04–40.29%) and caryophyllene oxide (7.74–40.13%), showed a strong antibacterial activity (MIC = 0.062−0.25 mg.mL^−1^) against *Rhodococcus equi* strains [63].

The EO from the leaves of *G. australis*, which is rich in germacrene B (50.6%), germacrene D (22.2%), and (*E*)-caryophyllene (8.9%), had little effect against *S. aureus* and *E. coli* (MIC = 250 µg.mL^−1^) [64].

The antimicrobial activities of EOs from the leaves, branches, and bark of *Bocageopsis pleiosperma* were evaluated. The EOs obtained from the bark had a moderate effect against *Staphylococcus epidermidis* (MIC = 250 µg.mL^−1^), while the other EOs did not show antimicrobial activity [58].

The EO from the leaves of *Annona vepretorum*, which is rich in bicyclogermacrene (43.7%), spathulenol (11.4%), α-phelandrene (10.0%), α-pinene (7.1%), (*E*)-β-ocimene (6.8%), germacrene D (5.8%), and *p*-cymene (4.2%), exhibited significant antimicrobial activity against *S. aureus*, *S. epidermidis*, and *Candida tropicalis*, with MIC values below 1000 µg.mL^−1^ [56].

The antimicrobial activities of EO oils from the leaves of *Annona pickelli*, which are rich in bicyclogermacrene (45.4%), (*E*)-caryophyllene (14.6%), and α-copaene (10.6%), and *Annona salzmannii*, with high contents of bicyclogermacrene (20.3%), (*E*)-caryophyllene (19.9%), δ-cadinene (15.3%), α-copaene (10.0%), and *allo*-aromadendreno (5.7%), were evaluated and the results obtained showed that the EO of *A. salzmannii* was more effective, exhibiting significant antimicrobial activity against most of the microorganisms tested [43].

The EO of *D. lanceolata*, which is rich in β-elemene (12.7%), caryophyllene oxide (12.4%), and β-selinene (8.4%), inhibited the growth of *Staphylococcus aureus*, *Streptococcus pyogenes*, *Escherichia coli*, and *Candida albicans*, with MIC values of 60, 20, and 60 µg.mL^−1^, respectively [38].

### 5.2. Anti-Inflammatory Activity

Many species of Annonaceae have been used to treat inflammatory diseases in folk medicine. Pharmacological studies have shown that some terpenoids and EOs from this family have significant anti-inflammatory effects, such as caryophyllene oxide and the EO of *Duguetia lanceolata*. The EO from the branches of *D. lanceolata*, which is rich in β-elemene (8.3%), β-caryophyllene (6.2%), caryophyllene oxide (7.7%), β-eudesmol (7.2%), β-selinene (7.1%), and δ-cadinene (5.5%), played a crucial role as a protective factor against carrageenan-induced acute inflammation [61].

The EO from the bark of the underground stem of *Duguetia furfuracea*, which is rich in (*E*)-asarone (21.9%), bicyclogermacrene (16.7%), 2,4,5-trimethoxystyrene (16.1%), α-gurjunene (15.0%), and cyperene (7.8%), was shown to have anti-inflammatory effects [13].

The EO from the leaves of *Annona sylvatica*, which are composed mainly of hinesol (8.16%), (*Z*)-caryophyllene (7.31%), β-maliene (6.61%), and γ-gurjunene (5.46%), showed anti-inflammatory activity against the persistent inflammation induced by CFA (Complete Freund’s Adjuvant) [45].

### 5.3. Antileishmanial Activity

The EO from the leaves of *Guatteria australis*, which has a high concentration of germacrene B (50.6%), germacrene D (22.2%), and (*E*)-caryophyllene (8.9%), presented anti-leishmania activity against *Leishmania infantum* (IC_50_ = 30.7 µg.mL^−1^) [64].

The EO of *Annona coriacea*, which has a high percentage of bicyclogermacrene (39.8%), presented antileishmania activity against the promastigote forms of four species of *Leishmania*, being more active against *L. chagasi* (IC_50_ = 39.93 µg.mL^−1^) [39].

### 5.4. Antioxidant Activity

Antioxidants are widely used in the food industry for a variety of reasons, including preventing oxidation; neutralizing free radicals; preserving food; and enhancing flavor, aroma, or color. As some synthetic antioxidants exhibit carcinogenic effects and can be toxic to nature, researchers have intensified the search for natural antioxidants [79]. In several studies with EOs, the antioxidant activity is related to compounds such as thymol, carvacrol, α-terpinene, β-terpinene, β-terpinolene, 1,8-cineol, eugenol, and linalool, which have an antioxidant activity similar to that of α-tocopherol [80].

The EOs from the leaves of two specimens of *Guatteria elliptica*, which were collected in Paranapiacaba and Caraguatatuba, showed a low antioxidant potential (EC_50_ = 7.24 and 28.68 mg.mL^−1^ using DPPH assays) for the EOs from Paranapiacaba and Caraguatatuba, respectively [9]. The difference in EC_50_ values can be attributed, at least in part, to the different contents of the main compounds present in EOs. Natural products such as EOs are formed by a complex mixture of organic compounds that act synergistically, increasing biological or even antagonistic activity and thus reducing the verified activity [80,81].

The EO of *Xylopia sericea* was investigated for its antioxidant potential using different methods. The EO of the fruit is rich in spathulenol (16.42%), guaiol (13.93%), and germacrene D (8.11%), and presented significant antioxidant activity through the DPPH (2,2-diphenyl-1-picryl-hydrazyl) methods (IC_50_ 49.1 μg·mL^−1^), β-carotene/linoleic acid bleaching (IC_50_ 6.9 μg·mL^−1^), TAC (Total Antioxidant Capacity) (IC_50_ 78.2 μg·mL^−1^), and TBARS (Thiobarbituric Acid Reactive Substances) (80.0 μg·mL^−1^) [50].

The EO from *Duguetia lanceolata* branches showed a high content of β-elemene (8.3%), β-caryophyllene (6.2%), caryophyllene oxide (7.7%), β-eudesmol (7.2%), β-selinene (7.1%), and δ-cadinene (5.5%). Antioxidant effects gained using the DPPH assay (EC_50_ 159.4 μg·mL^−1^), Fe^+3^ reduction (EC_50_ 187.8 μg·mL^−1^), and the inhibition of lipid peroxidation (41.5%) were considered significant [61].

The antioxidant potential of the EO of *Guatteria australis* leaves, which are rich in germacrene B (50.6%), germacrene D (22.2%), and (*E*)-caryophyllene (8.9%), was evaluated using two methods. Antioxidant capacity was considered either medium (TLC/DPPH, light yellow spot) or small (ORAC assay, 457 µmolTE.g^−1^) [64].

The EO from the leaves of *Annona vepretorum*, which are rich in bicyclogermacrene (43.7%), spathulenol (11.4%), α-phelandrene (10.0%), α-pinene (7.1%), (*E*)-β-ocimene (6.8%), germacrene D (5.8%), and *p*-cymene (4.2%), was able to capture radicals, but the antioxidant activity was considered weak. In the kinetic method of the ORAC assay, the result obtained was 204.24 µmolTE.g^−1^, while the TLC produced a yellow spot where the EO was applied due to the DPPH reduction [56].

The Eos from the leaves of *Annona pickelli*, which are rich in bicyclogermacrene (45.4%), (*E*)-caryophyllene (14.6%), and α-copaene (10.6%), as well as *Annona salzmannii*, which have high contents of bicyclogermacrene (20.3%), (*E*)-caryophyllene (19.9%), δ-cadinene (15.3%), α-copaene (10.0%), and *allo*-aromadendrene (5.7%), showed significant antioxidant capacity in the ORAC and DPPH assays [43].

### 5.5. Antiproliferative and Cytotoxic Activities

The search for new drugs that show activity against different types of cancer has become one of the most interesting subjects to research in the area of natural products. As a result, several EOs from Annonaceae species and their bioactive constituents were evaluated to determine their antiproliferative and cytotoxic properties.

The cytotoxic, mutagenic, and genotoxic profiles of the EO from *Xylopia laevigata* leaves were investigated. The results showed that the EO, which is rich in germacrene D (43.6%), bicyclogermacrene (14.6%), (*E*)-caryophyllene (7.9%), and germacrene B (7.3%), has mutagenic and antiproliferative activities, which can be related to the cytotoxic effect of the main components of the EO [76].

The in vitro cytotoxicity of *Annona vepretorum* EO (pure, microencapsulated with β-cyclodextrin and some of its main constituents) on tumor cell lines of different histotypes was evaluated. Furthermore, the in vivo efficacy of this EO in mice has been described. The results showed that the sesquiterpene spathulenol and EO, which have a high concentration of bicyclogermacrene (35.71%), spathulenol (18.89%), (*E*)-β-ocimene (12.46%), α-phellandrene (8.08%), and *o*-cymene (6.24%), exhibited promising cytotoxicity. The tumor growth in vivo was inhibited by EO treatment (34.46% inhibition) and EO microencapsulation was found to increase tumor growth inhibition (62.66% inhibition) [54].

The antitumor activity and toxicity of the EO of *Annona leptopetala* leaves, which are rich in spathulenol (12.5%) and α-limonene (9.0%), were evaluated. The EO showed antitumor activity in vitro and in vivo, mainly in the leukemia cell line, without major changes seen in the toxicity parameters evaluated [42].

The in vitro cytotoxic activity of the EO from fresh fruits of *Xylopia laevigata* and its main constituents (limonene, α-pinene, and β-pinene) was evaluated against four tumor cell lines (mouse melanoma, human hepatocellular carcinoma, human promyelocytic leukemia, and chronic myelocytic leukemia) and non-tumor cells (human peripheral blood mononuclear cells). Neither the EO nor its major constituents showed cytotoxic activity (IC_50_ > 25.0 µg.mL^−1^) [77].

The in vitro and in vivo antileukemic potential of the EO from the leaves of *Guatteria megalophylla* was investigated. The in vitro cytotoxic potential of the EO was evaluated in human cancer cell lines (HL-60, MCF-7 CAL27, HSC-3, HepG2, and HCT116) and in non-cancerous human cell lines (MRC-5). The in vivo efficacy was evaluated in C.B17 SCID mice with HL-60 cell xenografts. The results showed that this EO has anti-leukemic potential (with an IC_50_ value of 12.51 μg·mL^−1^ for HL-60 cells), and the main constituents spathulenol (27.7%), γ-muurolene (14.3%), bicyclogermacrene (10.4%), β-elemene (7.4%), and δ-elemene (5.1%) can play a central role in the registered activities [67].

The antiproliferative activity of the EO from the leaves of *Anaxagorea brevipes* was investigated in a number of cancer cell lines and the bioactivity was described against MCF-7 (breast, TGI = 12.8 μg·mL^−1^), NCI-H460 (lung, TGI) = 13.0 μg·mL^−1^), and PC-3 (prostate, TGI = 9.6 μg·mL^−1^). The antiproliferative activity found was attributed to the major constituents of the EO: β-eudesmol (13.16%), α-eudesmol (13.05%), γ-eudesmol (7.54%), and guaiol (5.12%) [51].

The antitumor activity and toxicity of *Xylopia langsdorffiana* EO, which is rich in α-pinene (34.5%) and limonene (31.7%), were evaluated. The EO was found to cause in vitro and in vivo growth inhibition in tumor cells, without major changes seen in the toxicity parameters evaluated [12].

The EOs from two specimens of *Guatteria elliptica* showed important antitumor activity against breast and prostate cancer cells (IC_50_ = 7.0 and 5.5 μg·mL^−1^, respectively) and a low cytotoxicity against normal fibroblasts (IC_50_ > 22.2 μg·mL^−1^ and IC_10_ = 18.5 μg·mL^−1^, respectively) [9].

The EO of *Duguetia gardneriana*, which has a high content of β-bisabolene (80.9%), exhibited a cytotoxic effect. The IC_50_ values were obtained for mouse melanoma, human hepatocellular carcinoma, human promyelocytic leukemia, and human chronic myelocytic leukemia cell lines (16.8, 19.1, 13.0 and 19.3 µg.mL^−1^, respectively). The in vivo antitumor activity was evaluated using C57BL/6 mice inoculated subcutaneously with B16-F10 melanoma cells, revealing tumor growth inhibition rates of 5.37 and 37.52% at doses of 40 and 80 mg/kg/day, respectively [11].

The antiproliferative activity of the EOs of four *Guatteria* species (*G. australis*, *G. ferruginea*, *G. latifolia*, and *G. sellowiana*) was investigated. These EOs contained the oxygenated sesquiterpenes spathulenol (11.04–40.29%) and caryophyllene oxide (7.74–40.13%) as the main constituents. The evaluation of the antiproliferative activity showed a strong selectivity (1.1–4.1 µg.mL^−1^) against the ovarian cancer tumor lineage, which was even more active than the positive control doxorubicin (11.7 µg.mL^−1^) [63].

The EO from the leaves of *Guatteria australis*, which had a high concentration of germacrene B (50.6%), germacrene D (22.2%), and (*E*)-caryophyllene (8.9%), had a strong antiproliferative effect against NCI-ADR/RES (ovarian- resistant) and HT-29 (colon). The TGI (Total Growth Inhibition) values were equal to 31.0 and 32.8 µg.mL^–1^, respectively [64].

The antiproliferative activity of the EO of *Annona sylvatica* leaves, which is rich in hinesol (8.16%), (*Z*)-caryophyllene (7.31%), β-maliene (6.61%), and γ-gurjunene (5.46%), was evaluated in vitro against nine human tumor cell lines: melanoma (UACC-62), breast (MCF-7), lung (NCI-H460), ovary (OVCAR03), prostate (PC-3), colon (HT-29), renal (786-0), resistant ovary (NCI/ADR-Res), and glioma (U251). The results demonstrate that the EO has anticancer activity, with GI_50_ values (concentrations that elicit an inhibition of 50% of the cell growth) in the range of 36.04–5.37 µg.mL^−1^, but at the highest concentration cytostatic activity and cytotoxic effects were observed for all cell lines [45].

The EO of *Annona pickelii*¸ which is mainly composed of bicyclogermacrene (38.0%), (*E*)-caryophyllene (27.8%), α-copaene (6.9%), and α-humulene (4.0%), as well as the EO of *Annona salzmannii*, which is rich in δ- cadinene (22.6%), (*E)*-caryophyllene (21.4%), α-copaene (13.3%), bicyclogermacrene (11.3%), and germacrene D (6.9%), exhibited potent antitumor activity. The most significant activity was observed against U251 (glioma, CNS), UACC-62 (melanoma), MCF-7 (breast), NCI-460 (lung), and HT-29 (colon) for the EO of *A. pickelii* e U251, 786-0 (kidney) and NCI-460 for the EO of *A. salzmannii*, all with TGI values below 50 µg.mL^−1^ [53].

*Xylopia laevigata* EO has significant anticancer potential in vitro and in vivo. The cytotoxic effects of EOs from the leaves of three specimens of *X. laevigata* were evaluated against different tumor lines: OVCAR-8 (ovarian carcinoma), SF-295 (GLIOBLASTOMA), HCT-116 (colon carcinoma), HL-60 (promyelocytic leukemia), and PBMC (peripheral lymphoblast). In the in vitro cytotoxic study, different EO samples with similar chemical profiles (γ-muurolene, δ-cadinene, germacrene B, α-copaene, germacrene D, bicyclogermacrene, and (*E*)-caryophyllene) showed cytotoxicity to all the tumor lines tested. In the in vivo antitumor study, the tumor growth inhibition rates were 37.3–42.5% [29].

The EO from *Xylopia sericea* leaves, characterized by α-pinene, β-pinene, *o*-cymene, and D-limonene, showed a low cytotoxicity to HepG2 cells (human hepatocellular carcinoma) (CC50 275.9 µg.mL^−1^) [78].

The EO from *Xylopia frutescens* leaves, which are rich in €-caryophyllene (31.48%), bicyclogermacrene (15.13%), germacrene D (9.66%), δ-cadinene (5.44%), viridiflorene (5.09%), and α- copaene (4.35%) showed cytotoxicity against the tumor cell lines NCI-H358M (bronchoalveolar carcinoma of the lung) and PC-3M (metastatic prostate carcinoma), with IC_50_ values ranging from 24.6 to 40.0 µg.mL^−1^, respectively. In the in vivo antitumor study, the tumor growth inhibition rates were 31.0–37.5% [33].

The antiproliferative activity of the EO from *Cardiopetalum calophyllum* leaves, which is mainly made up of spathulenol (28.78%), viridiflorol (9.99%), and (*Z*,*E*)-farnesol (6.51%), was evaluated in different human tumor cell lines: adenocarcinoma of the breast (MCF-7), cervical adenocarcinoma (HeLa), and glioblastoma (M059J), in addition to a normal human cell line (GM07492A, pulmonary fibroblasts). The IC_50_ values ranged from 216.8 to 353.51 µg.mL^−1^ and selectivity was not observed [82].

### 5.6. Larvicidal Activity

The larvicidal effect of EOs from Annonaceae species was tested against several disease vectors. The EOs of two species of *Duguetia* were evaluated against the larvae of *Artemia salina* and *Culex quinquefasciatus*. Essential oils from the leaf, wood, and bark of the underground stem of *D. furfuracea* showed potent activity against *A. salina* larvae (LC_50_ 6.01, 7.79 and 9.98 μg·mL^−1^, respectively). The main constituents were spathulenol (47.2%), bicyclogermacrene (26.4%), and caryophyllene oxide (5.2%) in the EO of the leaf, (*E*)-asarone (21.9%), bicyclogermacrene (16.7%), 2,4,5-trimethoxystyrene (16.1%), α-gurjunene (15.0%), and cyperene (7.8%) in the underground stem bark EO, as well as (*E*)-asarone (16.6%), cyperene (15.7%), spathulenol (14.2%), 2,4,5-trimethoxystyrene (13.2%), bicyclogermacrene (8.6%), and α-gurjunene (8.1%) in the wood EO. The EO of *D. lanceolata* leaves, which is rich in α-selinene (11.0%), aristolochene (5.8%), (*E*)-caryophyllene (5.3%), and (*E*)-calamenene (5.2%), also showed potent activity against *A. salina* larvae (LC_50_ 0.89 μg·mL^−1^). The EOs of both species were moderately active against *C. quinquefasciatus*, as they exhibited LC_50_ values ranging from 57.8 to 121.7 μg·mL^−1^ [60].

The EO of *Onychopetalum periquino*, which has a high concentration of β-elemene (53.16%), spathulenol (11.94%), and β-selinene (9.25%), showed a high larvicidal activity against *Aedes aegypti* larvae, with an LC_50_ of 63.75 µg.mL^−1^ reaching 100% mortality at 200 µg.mL^−1^ [70].

The EOs of *Xylopia laevigata*, which are rich in germacrene D (27.0%), bicyclogermacrene (12.8%), (*E*)-caryophyllene (8.6%), γ-muurolene (8.6%), and δ-cadinene (6.8%), and of *Xylopia frutescens*, which has high levels of bicyclogermacrene (23.2%), germacrene D (21.2%), (*E*)-caryophyllene (17.4%), β-elemene (6.3%), and (*E*)-β-ocimene (5.2%), did not show larvicidal activity [75].

The larvicidal activity of EOs from *Annona pickelli* leaves, which are rich in bicyclogermacrene (45.4%), (*E*)-caryophyllene (14.6%), and α-copaene (10.6%), and *Annona salzmannii*, which has high contents of bicyclogermacrene (20.3%), (*E*)-caryophyllene (19.9%), δ-cadinene (15.3%), α-copaene (10.0%), and *allo*-aromadendrene (5.7%), was determined against *Aedes aegypti* larvae. However, no larval mortality was detected at concentrations of up to 1000 µg.mL^−1^ [43].

The EO of *Duguetia lanceolata*, which is rich in β-elemene (12.7%), caryophyllene oxide (12.4%), and β-selinene (8.4%), was active against *A. salina* larvae with LC_50_ values equal to 49.0 μg·mL^−1^ and was about nine times more poisonous than the standard used thymol (LC_50_ = 457.9 μg·mL^−1^) [38].

The larvicidal activity of the EOs of *Guatteria blepharophylla*, *Guatteria friesiana*, and *Guatteria hispida* was tested against *A. aegypti* larvae; the lethal concentrations of LC_50_, LC_95_, and LC_99_ were, respectively, 85.74, 199.35, and 282.76 ppm for *G. hispida*; 58.72, 107.6, and 138.37 ppm for *G. blepharophylla*; and 52.6, 94.37, and 120.22 ppm for *G. friesiana*. The EO of *G. friesiana*, rich in α-, β- and γ-eudesmol, showed better insecticidal effect [65].

### 5.7. Trypanocidal and Antimalarial Activities

Chagas disease, also known as American trypanosomiasis, is caused by the protozoan parasite *Trypanosoma cruzi*. With a complex pathophysiology and dynamic epidemiological profile, this disease remains an important public health concern and is an emerging disease in non-endemic countries. For its etiological treatment in both the acute and chronic phase, there are two main drugs for the treatment of the disease: benznidazole and nifurtimox [83].

The EOs of *Bocageopsis multiflora*, *Duguetia quitarensis*, *Fusaea longifolia*, and *Guatteria punctata* were evaluated to determine their trypanocidal activity. The results showed that these EOs were active at the concentrations tested. The EO of *G. punctata* was the most active, with an IC_50_ = 0.029 μg·mL^−1^, being 34 times more active than the reference drug benznidazole. The authors reported that the strong activity observed for this species can be attributed to the presence of germacrene D (19.8%) and (*E*)-caryophyllene (8.4%) in the composition of the EO of *G. punctata* [57].

Essential oils extracted from the leaves of *Guatteria friesiana*, which have a high content of β-eudesmol (51.9%), γ-eudesmol (18.9%), and α-eudesmol (12.6%), and from the leaves of *Guatteria pogonopus*, which are rich in spathulenol (24.8%), γ-amorphene (14.7%), and germacrene D (11.8%), demonstrated potent trypanocidal and antimalarial activity with IC_50_ values below 41.3 μg·mL^−1^ [66].

The EO from the leaves of *Annona vepretorum*, which has high levels of bicyclogermacrene (43.7%), spathulenol (11.4%), α-phellandrene (10.0%), α-pinene (7.1%), (*E*)-β-ocimene (6.8%), germacrene D (5.8%), and *p*-cymene (4.2%), showed potent trypanocidal activity with an IC_50_ value equal to 31.9 µg.mL^−1^ [56].

The trypanocidal activity of EOs from *Annona pickelii*, which are rich in bicyclogermacrene (38.0%), (*E*)-caryophyllene (27.8%), α-copaene (6.9%), α-humulene (4.0%), and EOs from *Annona salzmannii*, which are rich in δ-cadinene (22.6%), (*E*)-caryophyllene (21.4%), α-copaene (13.3%), bicyclogermacrene (11.3%), and germacrene D (6.9%), were evaluated. The results showed that the *A. pickelii* EO was the most active, with an IC_50_ value of 27.2 µg.mL^−1^, while the IC_50_ value observed for *A. salzmannii* EO was 89.7 µg.mL^−1^ [53].

The EOs of *Annona squamosa*, which are rich in (*E*)-caryophyllene (27.4%), germacrene D (17.1%), and bicyclogermacrene (10.8%), and the EOs *Annona vepretorum*, which are rich in bicyclogermacrene (39.0%), spathulenol (14.0%), and α-phellandrene (11.5%), showed potent trypanocidal and antimalarial activity, with IC_50_ values below 20 μg·mL^−1^ and a strong inhibition of the proliferation of amastigote forms [44].

The EO of *Annona coriacea*, which has a high percentage of bicyclogermacrene (39.8%), showed trypanocidal activity against trypomastigote forms of *T. cruzi* (IC_50_ 168.50 µg.mL^−1^) [39].

The antiplasmodic activity of *Xylopia sericea* EO, which is characterized by α-pinene, β-pinene, *o*-cymene, and D-limonene, was evaluated and showed a low growth inhibition (24.0 to 50.0 µg.mL^−1^) against *Plasmodium falciparum*, a malaria-associated protozoan, in humans [78].

### 5.8. Other Activities

The EOs *of Xylopia laevigata* and *Xylopia frutescens* showed a low degree of protection against *Aedes aegypti* landings and, therefore, low repellent activity. The EO of *X. laevigata* had a high concentration of germacrene D (27.0%), bicyclogermacrene (12.8%), (*E*)-caryophyllene (8.6%), γ-muurolene (8.6%), and δ-cadinene (6.8%), while high levels of bicyclogermacrene (23.2%), germacrene D (21.2%), (*E*)-caryophyllene (17.4%), β-elemene (6.3%), and (*E*)-β-ocimene (5.2%) were identified in the EO of *X. frutescens* [75].

The anticonvulsant, sedative, anxiolytic, and antidepressant activities of the EO from the leaves of *Annona vepretorum*, which is rich in (*E*)-β-ocimene (42.59%), bicyclogermacrene (18.81%), germacrene D (12.19%), and limonene (10.02%), were investigated in mice. The results showed that acute treatment with the EO of this species has anxiolytic, sedative, antiepileptic, and antidepressant effects [55].

The sesquiterpene caryophyllene oxide and the EO from *Duguetia lanceolata* branches, which is rich in β-elemene (8.3%), caryophyllene oxide (7.7%), β-eudesmol (7.2%), β-selinene (7.1%), β-caryophyllene (6.2%), and δ-cadinene (5.5%), have an antinociceptive effect, as they were shown to reduce abdominal contortions in mice [61].

The insecticidal, antifungal, and antiaflatoxigenic activities of *Duguetia lanceolata* EO were evaluated in stored grain spoilage agents. The main constituents of this EO were β-bisabolene (56.2%) and 2,4,5-trimethoxystyrene (19.1%). The results suggested that the EO has promising grain protection properties against *Sitophilus zeamais* and *Zabrotes subfasciatus*, showing a comparable activity to that of a deltamethrin-based insecticide (positive control) [62].

The antinociceptive effect of a *Duguetia furfuracea* underground stem bark EO, composed mainly of (*E*)-asarone (21.9%), bicyclogermacrene (16.7%), 2,4,5-trimethoxystyrene (16.1%), α-gurjunene (15.0%), and cyperene (7.8%), was investigated. The results showed that the antinociceptive activity of this EO is possibly mediated by adenosinergic and opioidergic pathways and that its properties do not induce effects on motor coordination [13].

The EO from fresh leaves of *Unonopsis guatterioides*, which is rich in α-copaene (15.7%), bicyclogermacrene (15.7%), *trans*-caryophyllene (15.7%), α-humulene (9.0%), *allo*-aromadendreno (8.4%), and spathulenol (7.3%), showed a phytotoxic effect on the germination, growth, and development of monocotyledonous (*Allium cepa*) and dicotyledonous (*Lactuca sativa*) plants [72].

## 6. Methodology

In this work, a systematic review was carried out to show studies published between the years 2011 and 2021 on the chemical composition and biological properties of EOs of Annonaceae species collected in Brazil, which can serve as a reference for the future research and use of these species. In addition, a section on the ethnobotanical use of these species was also inserted in order to express their importance in traditional Brazilian medicine.

Pubmed, WOS, Scopus, and Scielo were used as virtual databases to search for the peer-reviewed articles that were used to compose the present work. The keywords used for the research were: “Annonaceae”, “óleos essenciais”, “essential oils”, “atividades biológicas”, “biological activities”, “ethnobotany”, and “medicinal use”.

The selection of manuscripts to compose this review was based on studies published in peer-reviewed journals; in addition, a careful review was carried out to confirm whether the species studied in the published articles were of Brazilian origin, as reported at www.floradobrasil.jbrj.gov.br (accessed on 29 September 2021). The quality of the reviewed studies is well known—only peer-reviewed articles were included, and we considered only papers in the English language for gathering data regarding the chemical composition and biological properties of EOs of Annonaceae species. However, for the section on the ethnobotanical use of these species, data published in the Portuguese language were also considered. Theses, Ph.D. dissertations, and unpublished articles were not included in this review. Therefore, we focused on phytochemical and/or in vitro, in vivo, and in animal studies, with the aim of providing up-to-date information on the biological properties of EOs from Annonaceae species collected in Brazil.

According to the website CrossRef, from 2013 to 2021, journal articles (1244), components (237), chapters (55), dissertations (32), posted content (10) peer reviews (3), datasets (3), conference papers (3), monographs (1), and books (1) were used, with the year 2018 (201) having the highest number of publications. The main journals that published articles on Annonaceae were ChemInform (53); Phytotaxa (43); Natural Product Research (27); Journal of Essential Oil Research (27); Botanical Journal of the Linnean Society (27); Blumea—Biodiversity, Evolution and Biogeography of Plants (27); Biochemical Systematics and Ecology (27); Nordic Journal of Botany (26); Kew Bulletin (25); and Taxon (24).

In the science direct database, a total of 1888 papers were published, including review and research articles, chapters, and books. The main periodicals were Journal of Ethnopharmacology (270); Biochemical Systematics and Ecology (49); Forest Ecology and Management (49); South African Journal of Botany (45); Phytochemistry (38); Phytochemistry Letters (34); Review of Palaeobotany and Palynology (33); Herbal Medicine (32); Industrial Crops and Products (28); Journal of Herbal Medicine (27); Flora (26); Molecular Phylogenetics and Evolution (23); Studies in Natural Products Chemistry (23); Asian Pacific Journal of Tropical Biomedicine (23); Food Research International (22); European Journal of Medicinal Chemistry (22); Tetrahedron Letters (21); Palaeogeography, Palaeoclimatology, Palaeoecology (21); Biomedicine & Pharmacotherapy (19); Dictionary of Trees, Volume 2: South America, 2014 (18); Brazilian Journal of Pharmacognosy (18); The Alkaloids: Chemistry and Biology (17); Phytom Medicine (16); Bioorganic & Medicinal Chemistry Letters (15); and Food Chemistry (14). By analyzing the numbers of papers published in the two databases, we were able to identify the importance of the topic for the scientific community. Furthermore, this is the first report on a literature review of the Annonaceae species found in Brazil.

## 7. Conclusions

Studies relating to natural products are important, as they can be sources of new chemically active molecules with potential applications in diverse human activities. In the present review, we note that Brazilian Annonaceae species can be sources of bioactive compounds such as α-pinene, β-pinene, limonene, (*E*)-caryophyllene, bicyclogermacrene, caryophyllene oxide, germacrene D, spathulenol, and β-elemene, which are present in the essential oils of the plants. Furthermore, the potential use of these EOs in terms of their antimicrobial, antiproliferative, cytotoxic, larvicidal, antioxidant, anti-inflammatory activities, etc., was also described. In some cases, it was possible to observe that the biological activity reported for the essential oil (EO) was superior to that of drugs available on the market, such as the EO of the species *Guatteria punctata*, which showed a trypanocidal effect that was 34 times more active than that of the reference drug benznidazole. This and other studies demonstrate that it is necessary to expand research to the EOs of Annonaceae, especially species occurring in Brazil, since studies on these are still scarce and there is a considerable number of Annonaceae species that are unexplored in terms of their content, chemical composition, and the biological activities of their EOs. In addition, the ethnobotanical use of some plants of this family was demonstrated, and it was found that the most cited species in folk medicine belong to the *Annona* genus.

## Figures and Tables

**Table 1:** Ethnobotanical use of Annonaceae species occurring in Brazil.

Scientific Name	Popular Name	Brazil Region	Part of the Plant Used	Medicinal Use	Reference
*Annona coriacea* (Mart.)	Marolo, araticum and araticum-liso	Southeast	Not specified	Parasites, ulcers, inflammatory processes, rheumatism and anthelmintic	[39]
*A. crassiflora* Mart.	-	Not specified	Fruits and seeds (infusion)	Diarrhea	[31]
*A. crassiflora*	Marolo	Cerrado	Seeds	Chronic diarrhea	[40]
*A. dioica* St. Hil.	Araticum	Cerrado	Seeds, fruit and leaves	Chronic diarrhea, emollient and rheumatism	[40]
*A. glabra* L.	-	Not specified	Leaves	Rheumatism	[31]
*A. glabra*	Araticum and araticum do brejo	Northeast	Leaves	Rheumatism and vermifuge	[41]
*A. leptopetala* (R.E.Fr) H. Rainer	Pinha-brava	Northeast	Not specified	Anti-tumor and anti-inflammatory	[42]
*A. montana* Macfad	Graviola, araticum-grande and jaca-do-Pará	Northeast	Leaves	Snake bites and obesity	[41]
*A. salzmannii* A. DC.	-	Northeast	Leaves and bark	Diabetes, tumors, and inflammation	[43]
*A. spinescens* Mart	-	Not specified	Fruits and seeds	Ulcers	[31]
*A. squamosa* L.	-	Northeast	Leaves	Stimulants, antispasmodics, sweats, anthelmintics, and insecticides	[44]
*A. squamosa*	-	Not specified	Leaves	Boils and ulcers	[31]
*A. squamosa*	Pinha, ata and fruta-de-conde	Northeast	Seeds	Bath to remove lice	[41]
*A. sylvatica* A. St.-Hil	Araticum, araticum-do-mato, cortiça and cortiça-amarela	Southeast	Leaves	Fever, cough, ulcers caused by syphilis, muscle spasms, and diarrhea	[45]
*A. vepretorum* Mart.	Pinha da Caatinga	Northeast	Roots and Leaves	Bee and snake stings, inflammation, heart pain, bath for allergies, skin diseases, fungal and bacterial infections	[46]
*Duguetia furfuracea* (A.St.-Hil.) Saff	Araticum-seco	Cerrado	Seeds	Parasiticidal	[47]
*D. furfuracea*	Araticum-seco	Southeast	Stem bark	Bath to remove lice	[41]
*D. furfuracea*	Araticum seco	Cerrado	Branches with leaves	Rheumatism	[40]
*D. lanceolata* St. Hil.	-	Not specified	Leaves	Anti-inflammatory	[31]
*D. lanceolata*	Pindaíba, beribá and pinhão	Not specified	Not specified	Anti-inflammatory, healing, and antimicrobial	[38]
*Guatteria ouregou* (Aubl.) Dunal.	-	North	Seeds	Dyspepsia, stomach and uterine pain	[36]
*Rollinia leptopetala* R.E.Fr	Pinha-brava	Northeast	Stem bark	Stomachic	[41]
*Xylopia aromatica* (Lam.) Mart.	Pimenta-de-macaco	Cerrado	Fruits, leaves and stem bark	Digestive and anti- inflammatory	[40]
*X. aromatica*	Pimenta-de-macaco, pimenta-de-negro	North	Not specified	Carminative and stimulating	[48]
*X. frutescens* Aubl.	-	Not specified	Barks	Flu	[49]
*X. frutescens*	Embira and semente-de-embira	Northeast	Seeds and fruits	Digestive	[41]
*X. frutescens*	Embira, embira-vermelha and pau carne	Northeast	Seeds	Bladder stimulant, triggering menstruation, fighting rheumatism, for halitosis, for tooth decay, and for intestinal diseases	[33]
*X. laevigata* (Mart.) R. E. Fries	Meiú and pindaíba	Northeast	Leaves and flowers	Painful disorders, heart disease, and inflammatory conditions	[34]
*X. sericea* A. St.-Hil.	Embiriba and pindaíba	Southeast	Seeds and fruits	Analgesic and anti-inflammatory	[50]
*X. sericea*	Pindaíba, pindaíba-vermelha and/or pimenta-de-macaco	Southeast	Not specified	Antimalarial	[35]

**Table 2:** Chemical composition of essential oils from Annonaceae species occurring in Brazil.

Species	Collection Place	Collection Date	Part of Plant (Yield)	Extraction Technique	Majority Constituents (% >5); Substance Classes; Total	Reference
*Anaxagorea Brevipes* Benth	Amazonas	September 2009	Leaves (0.52%)	HD	Guaiol, γ-eudesmol, β-eudesmol and α-eudesmol; M: 3.35%, SH: 13.56%; T: 75.69%	[51]
*Annona coriacea* Mart.	São Paulo	October 2009	Leaves (0.05%)	HD	Bicyclogermacrene, γ-muurolene and δ-cadinene; M: 20.0%, S: 76.7%; O: 3.3%; T: 96.5%	[39]
*A. exsucca* DC.	Pará	March 2019	Leaves (NI)	HD	Linalool, β-elemene, (*E*)-caryophyllene, α-humulene, germacrene D and bicyclogermacrene; HM: 2.3%, OM: 11.61%, SH: 80.52%, OS: 4.07%; T: 99.34%	[52]
*A. exsucca*	Pará	September 2019	Leaves (NI)	HD	*p*-Cymene, sylvestrene, terpinolene, linalool, germacrene D and bicyclogermacrene; HM: 43.36%, OM: 19.39%, SH: 31.29%, OS: 5.10%; T: 99.14%	[52]
*A. leptopetala* (R.E.Fr.) H. Rainer	Paraíba	August 2016	Leaves (0.04%)	HD	α-Limonene, linalool, α-terpineol, (*E*)-caryophyllene, bicyclogermacrene, spathulenol and guaiol; T: 98.1%	[42]
*A. pickelii* (Diels) H. Rainer	Sergipe	March 2010	Leaves (0.2%)	HD	Bicyclogermacrene, (*E*)-caryophyllene, α-copaene and germacrene D; M: 0.6%, S: 97.7%; T: 98.3%	[43]
*A. pickelii*	Sergipe	September 2010	Leaves (0.3%)	HD	Bicyclogermacrene, (*E*)-caryophyllene and α-copaene; T: 99.5%	[53]
*A. salzmannii* A. DC.	Sergipe	March 2010	Leaves (0.1%)	HD	Bicyclogermacrene, (*E*)-caryophyllene, δ-cadinene, α-copaene, and *allo*-aromadendrene; M: 2.5%, S: 93.7%; T: 96.2%	[43]
*A. salzmannii*	Sergipe	September 2010	Leaves (0.04%)	HD	δ-cadinene, (*E*)-caryophyllene, α-copaene, bicyclogermacrene and germacrene D; T: 98.7%	[53]
*A. squamosa* L.	Sergipe	September 2012	Leaves	HD	(*E*)-Caryophyllene, germacrene D and bicyclogermacrene; M: 2.0%; S: 65.1%; T: 99.1%	[44]
*A. sylvatica* A. St.-Hil Anelise	Mato Grosso do Sul	September 2010	Leaves (0.17%)	HD	Hinesol, (*Z*)-caryophyllene, β-malien, γ-gurjunene; T: 98.97%	[45]
*A. vepretorum* Mart.	Sergipe	April 2012	Leaves (0.59%)	HD	α-Phellandrene, *o*-cymene, (*E*)-β-ocimene, bicyclogermacrene and spathulenol; M: 30.18%, S: 67.41%, T: 97.59%	[54]
*A. vepretorum*	Pernambuco	January 2012	Leaves (0.09%)	HD	α-Pinene, limonene, spathulenol and caryophyllene oxide; T: 93.9%	[3]
*A. vepretorum*	Pernambuco	April 2015	Leaves (NI)	HD	Limonene, (*E*)-β-ocimene, germacrene D and bicyclogermacrene	[55]
*A. vepretorum*	Sergipe	April 2010	Leaves (NI)	HD	Bicyclogermacrene, spathulenol, α-phellandrene, α-pinene, (*E*)-β-ocimene, germacrene D and *p*-cymene; M: 29.2%, S: 68.9%; T: 98.1%	[56]
*A. vepretorum*	Sergipe	March 2012	Leaves (0.76%)	HD	Bicyclogermacrene, spathulenol and α-phellandrene; M: 34.0%; S: 65.1%; T: 99.1%	[44]
*Bocageopsis multiflora* (Mart.) R.E. Fr.	Amazonas	June 2013	Leaves (0.34%)	HD	α-*trans*-Bergamotene, β-bisabolene, spathulenol and β-copaen-4-α-ol; HM: 0.3%, OM: 1.0%, SH: 34.3%, OS: 49.5%, T: 95.0%	[49]
*B. multiflora*	Rondônia	July 2018	Aerial parts (0.12%)	HD	*cis*-Linalool oxide (furanoid) and 1-*epi*-cubenol	[57]
*B. pleiosperma* Maas	Amazonas	NI	Leaves (0.28%)	HD	(*E*)-α-Bergamotene, (*E*)-β-farnesene and β-bisabolene; T: 87.64%	[58]
*B. pleiosperma*	Amazonas	NI	Barks (0.27%)	HD	β-Selinene, α-selinene, β-bisabolene and δ-cadinene; T: 97.11%	[58]
*B. pleiosperma*	Amazonas	NI	Twigs (0.25%)	HD	β-Bisabolene, (2*Z*,6*Z*)-farnesol and cryptomerone; T: 72.64%	[58]
*Cardiopetalum calophyllum* (Schltdl.)	Goiás	September 2014	Flowers (NI)	HD	(*E*)-Caryophyllene, germacrene D and germacrene B; M: 0.51%, S: 70.11%	[59]
*C. calophyllum*	Goiás	December 2014	Fruits (NI)	HD	Germacrene D, germacrene B and spathulenol; M: 0.55%, S: 73.29%	[59]
*C. calophyllum*	Goiás	March 2014	Leaves (NI)	HD	Spathulenol, viridiflorol, (–)-isolongifolol acetate, and (*Z*,*E*)-farnesol; M: 0.43%, S: 66.04%	[59]
*Duguetia furfuracea* (A. St. -Hil.) Saff.	Minas Gerais	August 2016	Stem bark (0.5%)	SD	Cyperene, α-gurjunene, bicyclogermacrene, 2,4,5-trimethoxystyrene and (*E*)-asarone; T: 99.5%	[13]
*D. furfuracea*	Minas Gerais	August 2016	Leaves (0.8%)	HD	Spathulenol and bicyclogermacrene	[60]
*D. furfuracea*	Minas Gerais	August 2016	Underground parts (wood) (0.7%)	HD	(*E*)-Asarone, cyperene, 2,4,5-trimethoxystyrene, bicyclogermacrene and α-gurjunene	[60]
*D. furfuracea*	Minas Gerais	August 2016	Underground parts (trunk) (0.9%)	HD	(*E*)-Asarone and 2,4,5-trimethoxystyrene	[60]
*D. lanceolata* St. Hil.	Minas Gerais	April 2012	Twigs (0.4%)	HD	β-Elemene, β-caryophyllene, β-selinene, δ-cadinene, caryophyllene oxide, humulene II epoxide, β-eudesmol and cadina-1,4-dien-3-ol; HM: 4.0%, OM: 3.8%, SH: 40.0%, OS: 44.9%; T: 92.9%	[61]
*D. lanceolata*	Minas Gerais	NI	Leaves (0.4%)	HD	α-Selinene, aristolochene, (*E*)-caryophyllene and (*E*)-calamenene	[60]
*D. lanceolata*	São Paulo	March 2012	Leaves (0.3%)	HD	*trans*-Muurola-4(14),5-diene, β-bisabolene, 3,4,5- trimethoxy-styrene and 2,4,5-trimethoxy-styrene	[62]
*D. lanceolata*	Minas Gerais	NI	Barks (0.5%)	HD	β-elemene, caryophyllene oxide and β-selinene; HM: 1.6%, OM: 5.9%, SH: 31.9%, OS: 59.8%, H: 0.4%; T: 99.6%	[38]
*D. quitarensis* Benth.	Rondônia	June 2018	Aerial parts (0.11%)	HD	4-Heptanol, α-thujene, α-copaene, (*E*)-caryophyllene and germacrene D; M: 21.2%, OM: 2.5%, S: 37.8%, OS: 1.4%; T: 97.3%	[57]
*D. gardneriana* Mart.	Sergipe	November 2013	Leaves (0.13%)	HD	β-Bisabolene and elemicin; S: 96.0%; T: 96.0%	[11]
*Ephedranthus amazonicus* R.E. Fr	Amazonas	September 2012	Leaves (0.2%)	HD	Cyclosativene, α-muurolene, spathulenol, caryophyllene oxide and humulene epoxide II; OM: 0.6%, SH: 20.8%, OS: 74.2%; T: 98.0%	[49]
*Fusaea longifolia* Saff	Rondônia	July 2018	Aerial parts (0.18%)	HD	(*E*)-Caryophyllene, β-selinene, *cis*-β-guayene and (*Z*)-α-bisabolene; M: 0.1%, S: 85.6%, OS: 2.0%; T: 88.5%	[57]
*Guatteria australis* A. ST.-HIL.	Rio de Janeiro	February 2011	Aerial parts (0.1%)	HD	β-Pinene, *trans*-pinocarveol, *trans*-verbenol, myrtenol, spathulenol and caryophyllene oxide; M: 14.45%, OM: 27.47%, S: 0.76%, OS: 51.89%; T: 94.26%	[63]
*G. australis*	São Paulo	NI	Leaves (0.16%)	HD	(*E*)-Caryophyllene, germacrene D and germacrene B; M: 17.24%, S: 79.40%; T: 96.64%	[64]
*G. blepharophylla* Mart.	Amazonas	September 2012	Leaves (0.16%)	HD	Palustrol, spathulenol and caryophyllene oxide; SH: 6.4%, OS: 88.0%; O: 4.6%; T: 99.0%	[49]
*G. blepharophylla*	Amazonas	January 2008	Leaves (0.3%)	HD	Caryophyllene oxide; M: 0.1%, S: 91.2%; T: 91.3%	[65]
*G. elliptica* R. E. Fries	São Paulo	NI	Leaves (0.11%)	HD	Spathulenol and caryophyllene oxide; SH: 0.5%, OS: 99.5%; T: 100.0%	[9]
*G. elliptica*	São Paulo	NI	Leaves (0.21%)	HD	Spathulenol, caryophyllene oxide and β-copaen-α-ol; SH: 9.5%, OS: 91.5%, O: 0.5%; T: 100.0%	[9]
*G. friesiana* (W.A.Rodrigues) Erkens & Maas	Amazonas	NI	Leaves (1.17%)	HD	γ-Eudesmol, β-eudesmol and α-eudesmol; S: 93.0%; T: 93.0%	[66]
*G. friesiana*	Amazonas	January 2008	Leaves (0.6%)	HD	β-Eudesmol, γ-eudesmol e α-eudesmol; S: 98.2%; T: 98.2%	[65]
*G. hispida* (R.E. Fries)	Amazonas	July 2008	Leaves (0.5%)	HD	(*E*)-Caryophyllene; M: 68.4%, S: 31.0%; T: 99.4%	[65]
*G. latifolia* (Mart.) R.E.Fr.	Rio de Janeiro	February 2011	Aerial parts (0.1%)	HD	Spathulenol and caryophyllene oxide; OM: 6.94%, S: 3.35%, OS: 64.46%; T: 73.24%	[63]
*G. megalophylla* Diels	Amazonas	September 2018	Leaves (0.12%)	HD	δ-elemene, β-elemene, γ-muurolene, bicyclogermacrene and spathulenol; M: 1.41%, S: 87.30%; T: 88.71	[67]
*G. pogonopus* Mart.	Sergipe	NI	Leaves (0.22%)	HD	Germacrene D, γ-amorphene and spathulenol; S: 88.4%; T: 88.4%	[66]
*G. pogonopus*	Sergipe	February 2012	Leaves (0.28%)	HD	α-Pinene, β-pinene, (*E*)-caryophyllene, germacrene D, bicyclogermacrene and γ-patchoulene; M: 23.13%, S: 60.44%; T: 86.19%	[68]
*G. punctata* (Aubl.) R. A. Howard.	Rondônia	September 2018	Aerial parts (0.39%)	HD	(*E*)-Caryophyllene, germacrene D, *cis*-β-guayene and (*E*)-nerolidol; HO: 2.8%; M: 0.6%; S: 56.8%; OS: 19.1%; T: 79.3%	[57]
*G. sellowiana* Schltdl	Rio de Janeiro	February 2011	Aerial parts (0.1%)	HD	(*Z*)-β-Farnesene, β-bisabolene, *cis*-α-bisabolene, spathulenol and caryophyllene oxide; OM: 5.16%; S: 6.55%; OS: 78.28%; T: 89.99%	[63]
*G. ferruginea* A. St.-Hil.	Rio de Janeiro	February 2011	Aerial parts (0.1%)	HD	*trans*-Pinocarveol, myrtenol, (*E*,*E)-*α-farnesene, spathulenol and caryophyllene oxide; M: 1.47%; OM: 24.54%; S: 1.91%; OS: 60.41%; T: 88.33%	[63]
*Onychopetalum amazonicum* R.E.Fr.	Amazonas	March 2015	Leaves (0.18%)	HD	α-Copaene, (*E*)-caryophyllene, bicyclogermacrene, δ-cadinene, spathulenol and caryophyllene oxide; SH: 60.7%, OS: 27.1%; T: 87.8%	[69]
*O. amazonicum*	Amazonas	March 2015	Trunk bark (0.37%)	HD	α-Gurjunene, *allo*-aromadendrene and α-*epi*-cadinol; SH: 56.9%; OS: 35.3%; T: 92.2%	[69]
*O. amazonicum*	Amazonas	March 2015	Twigs (0.34%)	HD	α-Gurjunene, α-*epi*-cadinol and cyperotundone; SH: 27.5%; OS: 47.5%; T: 75.0%	[69]
*O. periquino* (Rusby) D.M. Johnson & N.A. Murray	Acre	March 2017	Leaves (0.24%)	HD	β-elemene, β-selinene and spathulenol; SH: 78.86%; OS: 12.45%; T: 91.31%	[70]
*Porcelia macrocarpa* R.E. Fries	São Paulo	NI	Leaves	HD	Germacrene D, bicyclogermacrene and phytol; M: 0.39%; S: 76.0%; D: 7.3%; T: 84.0%	[71]
*P. macrocarpa*	São Paulo	November 2011	Fruits	HD	Neril, geranil formate, γ-muurolene, δ-cadinene, dendrolasin, hexacosane; M: 44.8%; S: 37.1%; D: 0.51%; HC: 10.49%; O: 6.7%; T: 99.6%	[71]
*Unonopsis guatterioides* (A.DC.) R.E.Fr	Mato Grosso do Sul	March 2005	Leaves (0.15%)	HD	α-Copaene, β-elemene, (*E*)-caryophyllene, α-humulene, *allo*-aromadendrene, germacrene D, bicyclogermacrene and spathulenol	[72]
*Xylopia aromatica* (Lam.) Mart.	Amazonas	September 2012	Leaves (0.25%)	HD	*trans*-Pinocarveol, α-campholenal, camphor, dihydrocarveol, verbenone and spathulenol; HM: 2.2%; OM: 52.3%; SH: 14.6%; OS: 29.5%; T: 98.6%	[49]
*X. aromatica*	Goiás	February 2015	Leaves (0.1%)	HD	Bicyclogermacrene, spathulenol, globulol, *cis*-guaia-3,9-dien-11-ol and khusinol; OM: 2.74%, SH: 9.62%, OS: 71.25%, D: 1.2%, O: 13.15%; T: 97.96%	[73]
*X. aromatica*	Goiás	October 2014	Flowers (0.2%)	HD	Bicyclogermacrene, 7-*epi*-α-eudesmol, khusinol, pentadecan-2-one and *n*-tricosane; OM: 3.44%; SH: 17.24%; OS: 51.7%; D: 6.88%, O: 20.67%; T: 99.93%	[73]
*X. frutescens* Aubl.	Paraíba	April 2010	Leaves (NI)	HD	(*E*)-Caryophyllene, γ-cadinene, β-ocimene and cadin-4-en-10-ol; T: 90.20%	[74]
*X. frutescens*	Sergipe	April 2013	Leaves (NI)	HD	(*E*)-β-Ocimene, β-elemene, (*E*)-caryophyllene, germacrene D and bicyclogermacrene	[75]
*X. frutescens*	Sergipe	July 2011	Leaves (1.0%)	HD	(*E*)-Caryophyllene, bicyclogermacrene, germacrene D, δ-cadinene, viridiflorene and α-copaene; M: 0.41%; S: 96.10%; T: 96.51%	[33]
*X. laevigata* (Mart.) R. E. Fries	Sergipe	NI	Leaves (1.4%)	HD	Germacrene D, bicyclogermacrene, (*E*)-caryophyllene and germacrene B; T: 98.68%	[76]
*X. laevigata*	Sergipe	November 2012	Fresh fruits (0.4%)	HD	α-Pinene, β-pinene and limonene; M: 95.0%; S: 4.6%; T: 99.6%	[77]
*X. laevigata*	Sergipe	April 2013	Leaves	HD	(*E*)-Caryophyllene, γ-muurolene, germacrene D, bicyclogermacrene, δ-cadinene and germacrene B	[75]
*X. laevigata*	Sergipe	April 2010	Leaves (>1.0%)	HD	γ-Muurolene, δ-cadinene, germacrene D, bicyclogermacrene, α-copaene and (*E*)-caryophyllene; M: 2.14%, S: 95.35%; T: 97.49%	[34]
*X. laevigata*	Sergipe	March 2010	Leaves (1.58%)	HD	Germacrene D, bicyclogermacrene and (*E*)-caryophyllene; M: 1.15%, S: 98.60%; T: 99.75%	[34]
*X. laevigata*	Sergipe	July 2010	Leaves (>1.0%)	HD	Germacrene D, bicyclogermacrene, (*E*)-caryophyllene and germacrene B; M: 7.28%, S: 91.18%; D: 0.22 T: 98.68%	[34]
*X. langsdorffiana* St.-Hil. & Tul.	Paraíba	July 2012	Fresh fruits (0.03%)	HD	α-Pinene, camphene, D-limonene, caryophyllene oxide and esclarene; T: 100.0%	[12]
*X. sericea* A. St.-Hil.	Minas Gerais	September 2011	Fruits (0.93%)	HD	Germacrene D, spathulenol and guaiol; M: 9.65%; S: 81.5%; D: 7.79%; O: 0.1%; T: 99.04%	[50]
*X. sericea*	Minas Gerais	July 2012	Leaves (0.5%)	HD	α-Pinene, β-pinene, *o*-cymene and D-limonene	[78]

SD: steam distillation; HD: hydrodistillation; HC: hydrocarbons; D: diterpenes; M: monoterpenes (hydrocarbons and oxygenates); S: sesquiterpenes (hydrocarbons and oxygenates); HM: hydrocarbon monoterpenes; OM: oxygenated monoterpenes; SH: sesquiterpene hydrocarbons; OS: oxygenated sesquiterpenes; O: other class; NI: not informed; T: total identified compounds.
